# Domestic low temperature washing as a critical point in kitchen towel hygiene. Effectiveness of washing temperature, disinfectant and antibacterial finishing

**DOI:** 10.1186/s12866-026-05521-7

**Published:** 2026-08-12

**Authors:** Nina Čuk, Manca Lunder, Brigita Tomšič, Rok Fink

**Affiliations:** 1 University of Ljubljana, Faculty of Natural Sciences and Engineering, Aškerčeva 12, Ljubljana, 1000 Slovenia; 2 University of Ljubljana, Faculty of Health Sciences, Zdravstvena pot 5, Ljubljana, 1000 Slovenia

**Keywords:** Foodborne bacteria, Low temperature washing, Textile, Disinfectant, Antibacterial finishing

## Abstract

**Background:**

This study evaluated the efficacy of high- and low-temperature laundering, chemical disinfection, and antimicrobial textile finishes in reducing *Bacillus cereus*, *Salmonella enterica* serovar Typhimurium, and *Listeria monocytogenes* on domestic kitchen towels.

**Results:**

Polyester microfibre, cotton, and cotton/linen blend fabrics were experimentally inoculated and laundered with and without a commercial detergent at 60 °C–40 °C, treated with a commercial disinfectant, or subjected to an antibacterial finish. Laundering at 60 °C with detergent achieved reductions of more than 5 log₁₀ CFU/carrier for all tested bacteria, with complete reduction of S. enterica Typhimurium and L. monocytogenes across all textile types. In contrast, washing at 40 °C with detergent yielded reductions of less than 4 log₁₀ CFU/carrier and failed to effectively inactivate any of the target organisms, indicating that low-temperature laundering may constitute a significant reservoir for foodborne pathogens. Washing at 60 °C and 40 °C was less efficient than washing at a constant high temperature, indicating the combined effect of temperature and detergent. Both disinfectant treatments and the antimicrobial finish met established hygiene thresholds. Cross-contamination assessments revealed high bacterial transfer (more than 4 log₁₀ CFU/carrier) for all bacteria and textile types under baseline conditions, whereas laundering at 60 °C or applying the antimicrobial finish substantially reduced transfer. B. cereus, however, persisted following both mitigation approaches. Complete microbial inactivation on all textiles was achieved only with the addition of the disinfectant, underscoring its importance when lowering wash temperatures. Similar trends were observed in wash water: water from the 40 °C washing programme contained more than 4 log₁₀ CFU/mL, while water from the 60 °C washing programme and antimicrobial finishes limited survival primarily to B. cereus (detection limit 2.5 CFU/mL). The addition of disinfectant resulted in microbial reduction below the detection limit in wash water.

**Conclusions:**

Overall, low-temperature domestic laundering of kitchen towels may facilitate the dissemination of foodborne pathogens. *B. cereus* emerged as the least susceptible strain to both thermal and chemical interventions. Therefore, reductions in domestic wash temperatures should be accompanied by effective chemical disinfection to prevent foodborne illness transmission.

**Graphical Abstract:**

**Figure:**
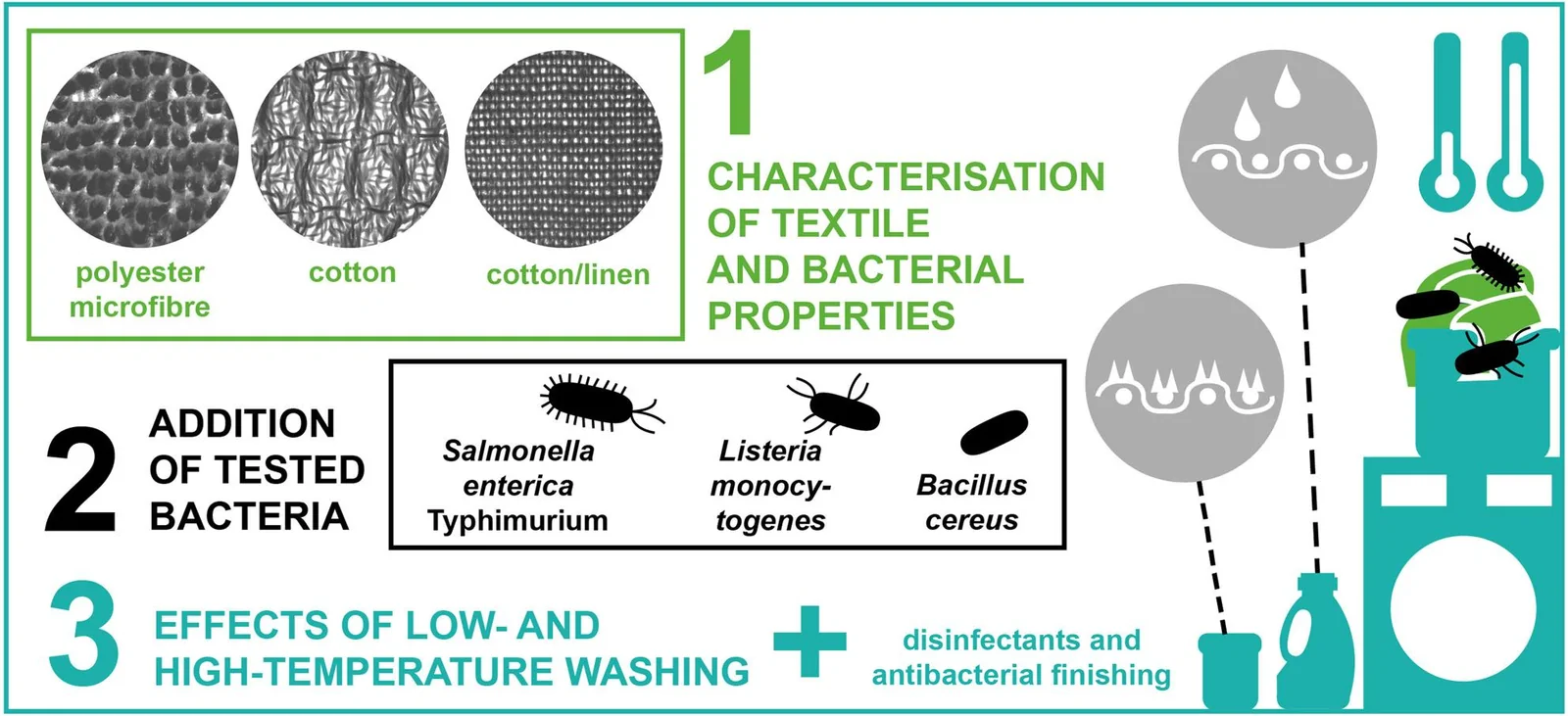

## Background

Kitchen towels play an important role in maintaining hygienically clean kitchen surfaces but may also act as vehicles for cross-contamination of pathogens from food spills to hands and other foods [1, 2]. EFSA reports a significant number of cases in domestic kitchen environments, with more than 5,000 outbreaks, 17,500 cases, 3,500 hospitalisations and 28 deaths recorded worldwide between 2020 and 2024. Among the causes, *Salmonella* spp. was most frequently reported, followed by unknown sources and unknown sources of bacterial toxins. In cases of mortality, the leading cause was *Salmonella* spp., followed by unknown sources and *L. monocytogenes* [2]. Current evidence suggests that microbial hotspots in domestic kitchens, including sinks, fridges and cleaning tools, harbour complex ecosystems of bacteria that can be transmitted via various surfaces, including kitchen textiles [3, 4]. The interaction of microorganisms with textiles depends on various factors, such as the type and strain of microorganism, surface characteristics of the microbe, and environmental factors including moisture availability, nutrients and temperature [5, 6]. Microbial adhesion to textiles is also influenced by material surface characteristics such as chemical composition, roughness, porosity and surface charge [7]. Laundry washing helps prevent the spread of microorganisms in the domestic environment by removing potential pathogens from textiles [8]. However, recent efforts to improve energy efficiency in laundry have led to a continuous decrease in washing temperatures. Government regulations and various initiatives encourage reduced energy and water usage, promoting the widespread adoption of low-temperature washing cycles at 30–40 °C [9–11]. This shift creates a paradox: while low-temperature washing supports environmental goals, it may compromise the hygiene safety of laundered textiles. Multiple studies confirm that washing at 30 °C is insufficient for complete microbial inactivation [12–15]. When washing temperature is reduced, compensation should be made through longer washing cycles, additional chemicals or increased mechanical activity to achieve sufficient bacterial reduction [16]. Chlorine- or oxygen-based antimicrobials, quaternary ammonium or phenolic compounds, organic acids or enzymes are added to laundry detergents to achieve further reduction [17–19]. Furthermore, metal-based coatings, organic and polymeric antibacterial coatings, and natural and photocatalytic finishes can be applied to prevent bacterial colonisation on textiles [20–22]. Although various studies have demonstrated the questionable efficacy of low-temperature washing, a comparative analysis of different conditions – such as temperature, detergent and disinfectant – on different textile types and hygienically relevant bacteria is still lacking.

The aim of this study was to evaluate the hygienic efficacy of different laundry approaches for kitchen towels (polyester microfibre, cotton and cotton/linen) by assessing the effects of high- (60 °C) and low-temperature (40 °C) washing, as well as the use of disinfectants and antibacterial coatings, on the reduction of *S. enterica* Typhimurium, *L. monocytogenes* and *B. cereus*. More specifically, the goal was to assess bacterial reduction under low- and high-temperature washing, with and without detergent, in order to elucidate the mechanisms of thermal and chemical disinfection underlying this reduction. Furthermore, the study examined how the addition of disinfectant or antibacterial finishing could compensate for the lower temperature while still meeting hygiene standards.

## Materials and methods

### Textile material, test organisms and chemicals used

Three commercially available kitchen towel materials were included in the research. The first was made of microfibre (80% polyester, 20% polyamide) in a terry weave with warp pile loops, measuring 40 × 40 cm. The second was 100% cotton in a waffle weave (50 × 70 cm), and the third was a cotton/linen blend in a plain weave (50 × 70 cm). All three textile samples were washed before testing using a gentle wash programme (800 rpm, 30 °C, 49 min), neutralised with 15 mL/L acetic acid, rinsed in the washing machine to ensure a neutral substrate, and finally completely air dried before further analysis. A commercially available domestic liquid detergent for white laundry, containing 5–15% anionic surfactant components, less than 5% non-ionic active components, phosphonates, soap, enzymes, benzothiazolinone, alpha-iso-methyl-ionone, citronellol, geraniol and linalool, was used in this study.

A commercial disinfectant for household laundry was tested, containing didecyldimethylammonium chloride (DDAC) as the active component at 1.8 g/100 g. It also contains cationic surfactants (less than 5%), methylisothiazolinone, phenoxyethanol, 2-bromo-2-nitropropane-1,3-diol, and non-ionic surfactants (less than 5%).

To impart antibacterial activity, the kitchen towels were additionally treated with the finishing agent Si Bac-Pure^®^ Antimicrobial 101-TF (Smart Innovation, Portugal), which contains benzalkonium chloride as the active compound. A 10 g/L aqueous solution of the finishing agent was prepared for application. The finishing process involved fully immersing the samples in the finishing bath approximately 60 s to ensure complete wetting, followed by padding using a two-roller foulard (Mathis, Switzerland) to achieve a wet pick-up of 80 ± 5%. The samples were then dried for 1 min in a laboratory continuous hot-air dryer (KTF 500, Mathis, Switzerland) and cured at 150 °C for 5 min.

For this study, reference strains of *B. cereus* ATCC 11,778, *S. enterica* Typhimurium CCM 3807 and *L. monocytogenes* ATCC 19,115 were obtained from the collection of microorganisms at the Faculty of Health Sciences, University of Ljubljana. The cryogenically preserved bacteria were plated on non-selective Tryptic Soy Agar (TSA) (Sigma-Aldrich, India) and incubated for 24 h at 37 °C before analysis.

### Determination of the textile and bacterial properties

The morphological characteristics of the textile samples were examined by scanning electron microscopy (SEM) using a JEOL JSM 6060 LV microscope (JEOL, Japan). The samples were observed in low vacuum at various magnifications: the surface of the textile samples at 22× magnification and the yarns at 1500× magnification. Before examination, all samples were coated with a thin layer of gold to increase conductivity. Further visual analysis of surface openness was performed using a stereomicroscope (Leica Microsystems GmbH, Germany) at 6× magnification. The openness of the textile sample surfaces was then analysed using the ImageJ programme.

The following physical properties of the textile samples were evaluated: thickness, mass per unit area, and air permeability. Thickness was measured according to the SIST EN ISO 5084 standard, with an applied load of 20 cN/cm² and a measuring foot area of 25 cm². Ten measurements were taken for each sample, and the average value is reported. Mass per unit area was determined according to the SIST EN 12,127 standard. Samples were cut to 10 × 10 cm and weighed on a gram scale. Five measurements were taken for each sample, and the average value is reported. Air permeability was measured using the Air-Tronic B air permeability tester (MESDAN, Italy) in accordance with the SIST EN ISO 9237 standard. The air volume was set to 10 L, the air pressure to 100 Pa, and the test area to 20 cm². For each fabric sample, ten measurements were taken at different locations.

The surface roughness of the textile samples was evaluated using a LEXT OLS5000 confocal laser scanning microscope (CLSM; Olympus, Tokyo, Japan). The scanned area was 2560 μm × 2560 μm at 5× magnification. A non-contact three-dimensional (3D) laser microscopy assessment was performed using a laser light source with a wavelength of 405 nm and a maximum lateral resolution of 0.12 μm. The average absolute deviation of roughness irregularities from the mean sample surface over the sampling area (Sa) was calculated using OLS50-S-AA software (Olympus, Tokyo, Japan). In addition to Sa values, surface topography was evaluated using the skewness of surface heights (Ssk) and the kurtosis of surface heights (Sku).

The chemical properties of the textile samples were analysed by Fourier transform infrared spectroscopy (FTIR). Spectra were recorded with a Spectrum 3 spectrometer (PerkinElmer, UK), using an ATR cell with a diamond crystal in the wavelength range 4000–500 cm⁻¹. The results were processed using the Spectrum IR software.

Moisture content, based on the physical and chemical properties, was determined gravimetrically using a moisture analyser (MLB-C, Kern & Sohn GmbH, Germany). Samples were equilibrated at 20 °C and 65 ± 2% relative humidity for 24 h prior to analysis. Drying was performed at 100 °C until a constant mass was achieved. Moisture content was calculated as the relative mass loss with respect to the initial mass of the conditioned sample. Three measurements were taken for each individual kitchen towel.

The hydrophobicity of the bacterial cells was determined by the MATH test (microbial adhesion to hydrocarbons): the bacterial culture grown overnight on TSA was diluted in phosphate-buffered saline (PBS; 1.15 g Na₂HPO₄, 0.2 g KH₂PO₄, 8 g NaCl, 0.2 g KCl per litre), centrifuged at 5000 g for 4 min, and washed twice. The cells were then resuspended in 20 mL PBS, and the absorbance was measured with a spectrophotometer (Nanocolour VIS, Germany) at a wavelength of 620 nm. Subsequently, 0.5 mL xylene (Carlo Erba, Italy) was added to 3.5 mL of the bacterial suspension in PBS, and the mixture was vortexed for 1 min at a maximum speed of 2500 rpm. After separation of the two layers (15 min), the optical density of the aqueous phase was measured. The bacterial hydrophobicity index was calculated by measuring the optical density of the suspension before and after partitioning with xylene and expressing the proportional decrease in aqueous-phase absorbance relative to the initial PBS suspension as a percentage.

### Assessment of bacterial reduction by low- and high-temperature washing, disinfection, and antibacterial finishing

The efficiency of bacterial washing was tested according to the standard SIST EN 17658:2021 as follows. A bacterial working culture was prepared by spreading on Tryptic Soy Agar and incubating at 37 °C for 24 h. The bacteria were transferred from solid medium to 0.9% NaCl, and the concentration was adjusted using a spectrophotometer at 620 nm. The suspension was then centrifuged at 4700 g for 5 min, the supernatant was removed, and the pellet was resuspended in 0.3% bovine serum albumin (BSA) solution. Autoclaved textile carriers were inoculated with 30 µL of the individual bacterial suspension in BSA (single culture) using a pipette, applying the suspension directly to the surface of the 1 × 1 cm samples, and then dried in a safety cabinet for 30 min. The samples were washed in a Starlet-2 infrared staining machine (Daelim Starlet Co., Ltd., Korea). Each experiment included five contaminated carrier samples for each micro-organism, five uncontaminated carriers for cross-contamination assessment, and five contaminated carriers that were not treated and served as controls. A 500 mL metal canister containing 36 g of ballast (65% polyester/35% cotton) and four steel beads was autoclaved. The interfering substance Soil Ballast Load (SBL) 2004 (4 g) was added to the canister. The commercial detergent was diluted to a concentration of 1.375 mL/L in standard hard water to obtain 400 mL of wash water. The detergent dosage was determined in accordance with the manufacturer’s instructions, corresponding to 55 mL of detergent per 4 kg of laundry. Washing tests at 60 °C and 40 °C were carried out without detergent to assess the effect of temperature alone.

In the next test, either 0.15% commercial disinfectant DDAC or antibacterial-finished carriers of the studied kitchen towels were used in combination with detergent at 40 °C. The washing time was 30 min and the washing temperature 40 °C. The concentration of DDAC was set according to the manufacturer’s recommendations, corresponding to 120 mL of disinfectant for a standard domestic washing machine with a drum capacity of 8 kg. No rinsing was carried out. Immediately after the wash cycle, the textile carriers were transferred to microtubes containing 1 mL neutralising agent (lecithin 3 g/L, polysorbate 80 30 g/L, sodium thiosulphate 5 g/L, saponin 30 g/L in PBS). The tubes were mixed with a Plate Shaker LLG at 18 °C and 1,500 rpm for 10 min. The samples were then serially diluted and 0.4 mL of each sample was inoculated onto Tryptic Soy Agar (limit of detection 2.5 CFU/carrier). Twenty millilitres of wash water was aspirated with a syringe and neutralised with 20 mL neutraliser at double concentration. After that, 1 mL of the sample was inoculated onto TSA (limit of detection 1 CFU/carrier). The samples were incubated at 37 °C for 24 h. The number of colonies was then counted. The controls were textile carriers with selected strains without washing. The bacteria on the control carriers were treated in the same way as the samples. The initial bacterial load on the carriers was, for *B. cereus*, 7.6 ± 0.3 log_10_CFU/carrier for microfibre, log_10_7.4 ± 0.4 CFU/carrier for cotton and 7.4 ± 0.4 log_10_CFU/carrier for cotton/linen; for *S. enterica* Typhimurium, 8.6 ± 0.2 log_10_CFU/carrier for microfibre, 7.8 ± 0.2 log_10_CFU/carrier for cotton and 8.0 ± 0.1 log_10_CFU/carrier for cotton/linen; and for *L. monocytogenes*, 7.8 ± 0.1 log_10_CFU/carrier for microfibre, 7.6 ± 0.3 log_10_CFU/carrier for cotton and 7.7 ± 0.2 log_10_CFU/carrier for cotton/linen. The reduction (log_10_ CFU/carrier) was calculated relative to the control.

### Statistical analysis

R version 4.2.2 (R Core Team, 2021) was used for statistical analysis. Normality was assessed using the Shapiro–Wilk test (*p* > 0.05). Duncan’s test and one-way analysis of variance (ANOVA) were used to determine significant differences. The significance level was set at *p* < 0.05. All experiments were conducted with three parallel trials and three replicates.

## Results

### Determination of the textile and bacterial properties

**Fig. 1:**
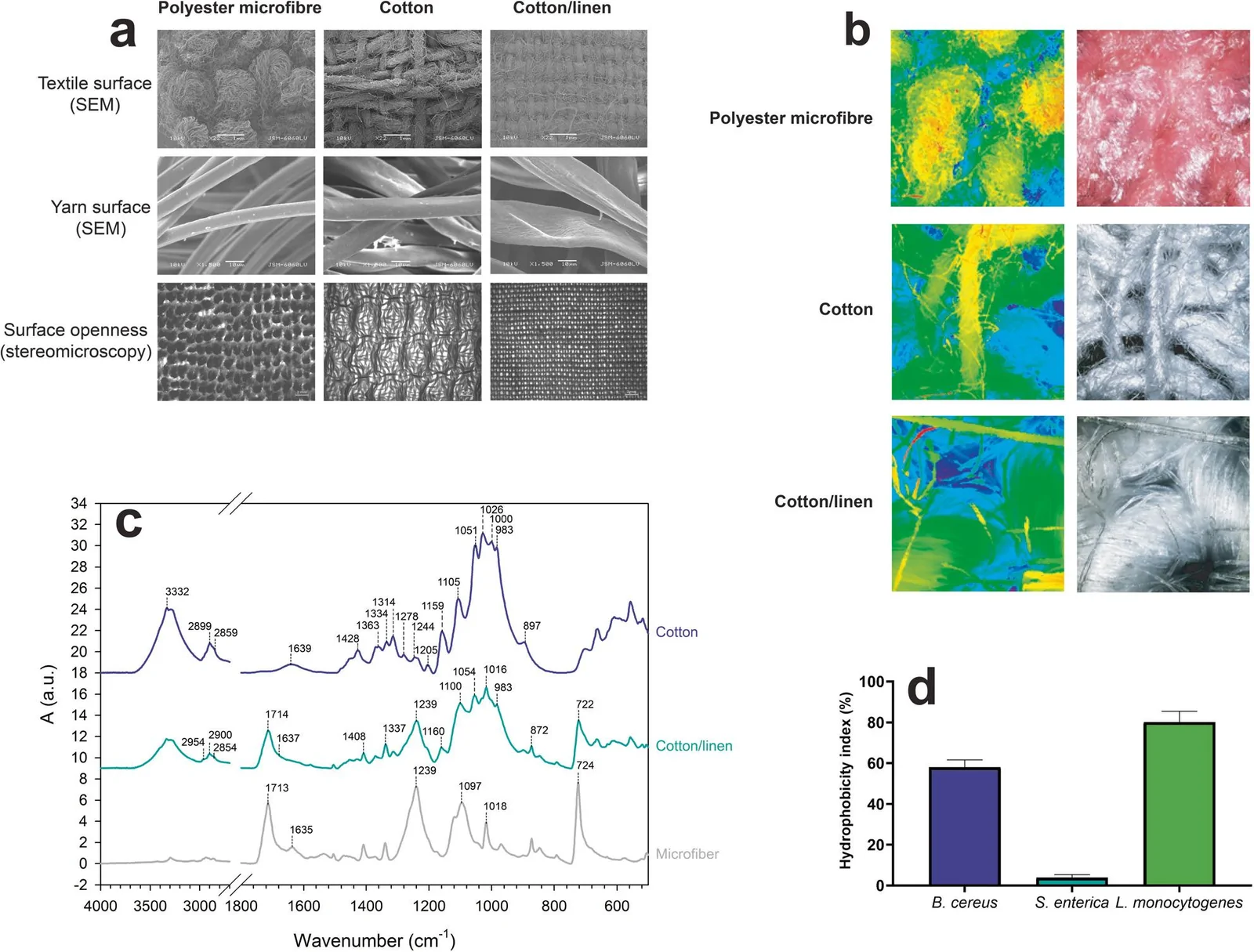
Characterisation of textile samples by (**a**) SEM, (**b**) CLSM, (**c**) FTIR and (**d**) bacterial cells by MATH

Although all three kitchen towels were produced using the same textile manufacturing technique (weaving), pronounced differences in surface morphology were observed among the materials, as revealed by SEM analysis (Fig. 1a). The cotton and cotton/linen samples exhibit visually similar surface characteristics, appearing more compact and predominantly two-dimensional. In contrast, the microfibre sample displays a more three-dimensional topography, characterised by pronounced surface undulations and volumetric features. At the yarn level, the cotton and cotton/linen samples show characteristic folds and surface irregularities, reflecting their intrinsic fibrillar structure and lower packing density. The microfibre sample, although synthetic and more uniform and smooth in composition, also exhibits surface irregularities, which can be attributed to the presence of matting agents added during fibre production to reduce surface gloss. These agents induce controlled roughening, resulting in a less reflective and more textured surface.

The surface openness results also show similarity between the cotton and cotton/linen samples (12.501% and 13.636%, respectively), while the microfibre sample has substantially lower openness (6.618%), indicating a denser, more compact structure (Table 1). Nevertheless, the cotton (1.470 mm) and microfibre (1.550 mm) fabrics have distinct thickness values, while the cotton/linen blend is significantly thinner (0.565 mm). These findings reflect the influence of weave architecture and fibre morphology: the plain weave of the cotton/linen sample typically produces a relatively flat, uniform surface, as does the waffle weave of the cotton sample, whereas the microfibre fabric is characterised by a highly textured, three-dimensional morphology resulting from densely packed microfilaments arranged in looped structures. In addition, the differences in average thickness are greater in the cotton and cotton/linen textile samples, indicating greater variability in surface structure, while the microfibre fabric appears to have a more stable engineered topography. The mass per unit area further differentiates the materials, following the trend: cotton/linen < cotton < microfibre. This observation correlates well with the SEM findings, in which the microfibre fabric shows a higher density of fine filaments compacted into three-dimensional surface features. The cotton fabric exhibits the greatest variability in areal mass, likely due to the greater flexibility of its yarns, allowing local rearrangements and variations in packing density. Finally, the textile samples also exhibit different air permeabilities, which are consistent with the structural differences. The cotton/linen and microfibre samples again have very similar values (60.0 and 66.5 cm³/(cm²·s), respectively), while the cotton sample allows the highest airflow through the sample, with a value of 181.3 cm³/(cm²·s).

**Table 1:** Characteristics of textiles

Textile analysis/ Sample	Polyester microfiber	Cotton	Cotton/Linen
Surface openness (%)	6.618	12.501	13.636
Thickness (mm)	1.550 ± 0.007	1.470 ± 0.033	0.565 ± 0.020
Mass per unit area (g/m^2^)	263.95 ± 1.05	202.07 ± 3.03	178.72 ± 0.78
Air permeability (cm^3^/(cm^2^*·*s))	66.5 ± 0.6	181.3 ± 1.5	60.0 ± 1.5
S_a_ (µm)	475.533	241.179	52.557
S_sk_ (/)	-0.661	-0.293	0.004
S_ku_ (/)	2.847	3.069	3.228
Moisture content (%)	2.54 ± 0.52	6.60 ± 0.08	10.69 ± 0.83

Surface roughness analysis (Fig. 1b; Table 1) using CLSM further clarifies the macroscopic topographical differences between the materials. The arithmetic mean surface height (Sa) increases in the order cotton/linen (52.557 μm) < cotton (241.179 μm) < polyester microfibre (474.533 μm), confirming that the microfibre textile has the greatest overall surface roughness. In addition to Sa, surface skewness (Ssk) values show that the cotton/linen surface is dominated by protruding peaks (Ssk > 0), whereas the cotton and microfibre surfaces are characterised by a prevalence of valleys (Ssk < 0), indicating recessed surface features. Surface kurtosis (Sku) further characterises the shape of the height distribution. Cotton and cotton/linen exhibit Sku values greater than 3, indicating sharp asperities or deep surface depressions. In contrast, the polyester microfibre has an Sku value below 3, suggesting broader, more rounded surface features with fewer extreme height variations. This is consistent with its highly textured three-dimensional morphology, which reflects the complex fibre architecture rather than sharp individual surface asperities.

To confirm the material composition of the kitchen towels investigated, FTIR spectroscopy was performed (Fig. 1c). Clear spectral differences between natural and synthetic fibres were observed. The most distinctive feature of polyester microfibre is the intense absorption band at 1713–1715 cm⁻¹, corresponding to the C = O stretching vibration of ester groups, characteristic of polyester-based materials. Additionally, pronounced aromatic ring vibrations appear around 724 cm⁻¹, further confirming the synthetic origin of the microfibre sample. In contrast, cellulose-based textiles (cotton and cotton/linen) display a broad absorption band centred at approximately 3330 cm⁻¹, attributed to O–H stretching vibrations and indicative of the abundant hydroxyl groups present in cellulose. Furthermore, strong absorption bands between 1200 and 900 cm⁻¹ correspond to C–O stretching and glycosidic linkages, while the band near 1635 cm⁻¹ is associated with absorbed water molecules, a typical feature of hydrophilic natural fibres.

These chemical differences are directly reflected in the moisture content of the textiles studied (Table 1). The highest moisture uptake was observed for the cotton/linen fabric, with an average moisture content of 10.69 ± 0.83%, due to the combined presence of cellulose and linen fibres, both rich in hydrophilic hydroxyl groups. The cotton sample exhibited moderate moisture absorption (6.60 ± 0.08%), consistent with its purely cellulosic composition but more compact structure. Thus, the presence of hydroxyl-rich cellulose promotes water sorption through hydrogen bonding, whereas the ester-dominated structure of polyester restricts moisture absorption, resulting in significantly lower moisture uptake (2.54 ± 0.52%). This is characteristic of synthetic fibres with low polarity and limited hydrophilic functional groups.

Beyond the characterisation of textile properties, bacterial surface hydrophobicity or hydrophilicity, which substantially influences how bacteria interact with surfaces, including textiles, can be quantified using the hydrophobicity index. In this study, the highest index was observed for *L. monocytogenes* (80%), followed by *B. cereus* (60%), indicating the hydrophobic nature of these two strains, while *S. enterica* Typhimurium was more hydrophilic (5%) (Fig. 1d). 

### Assessment of bacterial reduction by low- and high-temperature washing, disinfection, and antibacterial finishing

**Fig. 2:**
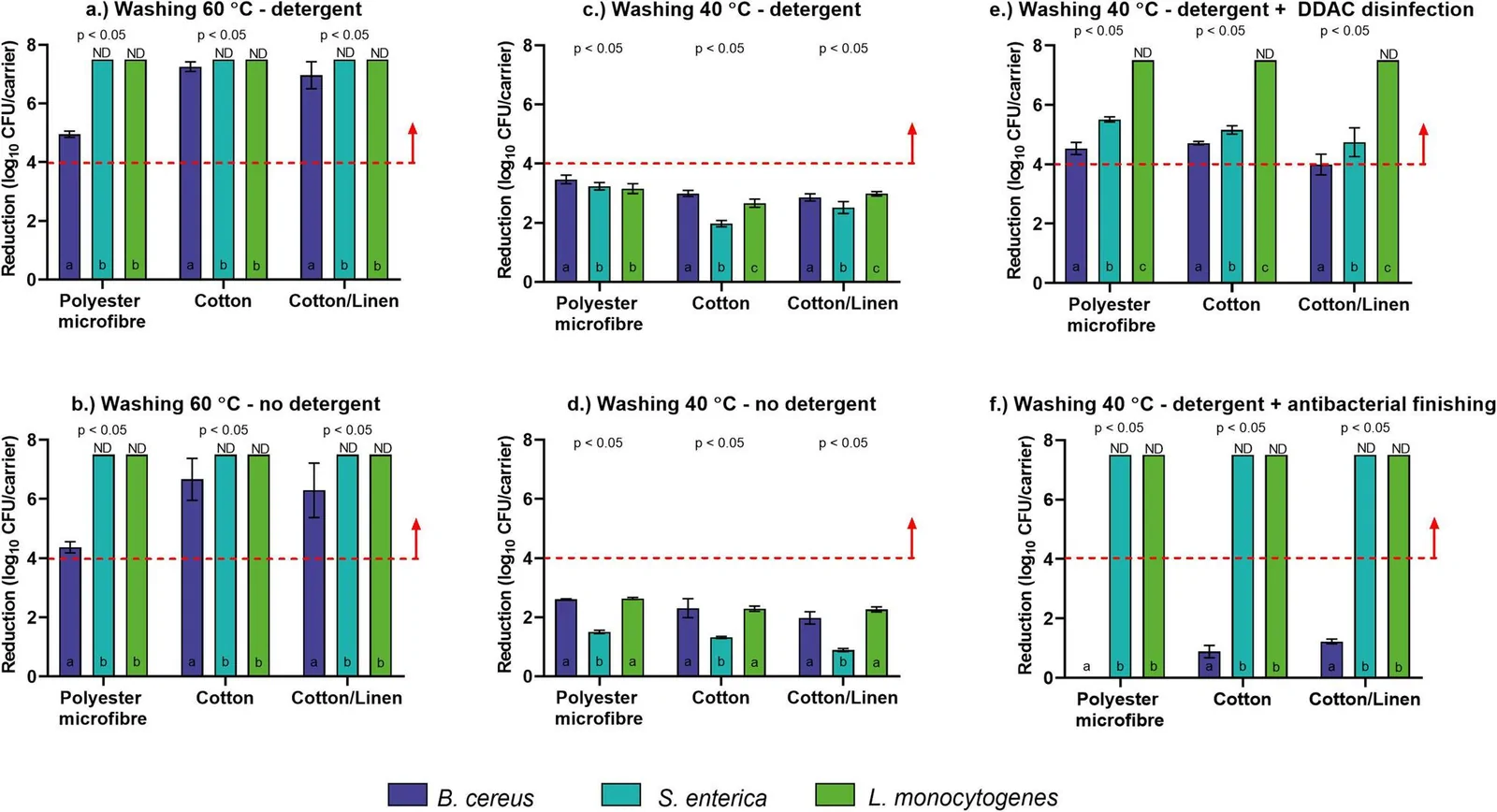
Reduction of *B. cereus*, *S. enterica* Typhimurium, and *L. monocytogenes* (log10 CFU/carrier) on polyester microfibre, cotton, and cotton/linen after (**a**) washing with detergent at 60 °C, **b** washing without detergent at 60 °C, **c** washing with detergent at 40 °C, **d** washing without detergent at 40 °C, **e** washing at 40 °C with detergent and DDAC disinfectant, and **f** washing antibacterial-finished samples with detergent at 40 °C. ANOVA and Duncan's post hoc test. Different letters above the columns (**a**–**c**) indicate significant differences in Duncan's test (*p* < 0.05). ND – not detected. The red dashed line represents the ISO 17658 standard threshold for successful laundry disinfection (≥ 4 log10 CFU/carrier)

Washing with commercial detergent at 60 °C reduced *S. enterica* Typhimurium and *L. monocytogenes* on polyester microfibre, cotton, and cotton/linen to below the detection limit. However, a lower reduction was observed for *B. cereus*, although a reduction of more than 4 log₁₀ CFU/carrier was still achieved (Fig. 2a). A similar trend was observed for washing at 60 °C without detergent, although the reduction was lower than with detergent washing (Fig. 2b).

In contrast, washing at 40 °C with commercial detergent did not meet hygiene requirements for any of the tested bacteria or textiles. The highest reduction was observed for polyester microfibre, followed by cotton/linen, with the lowest reduction for cotton (Fig. 2c). A similar trend was seen for washing at 40 °C without detergent, although the reduction was lower than with detergent washing (Fig. 2d). Washing at 40 °C with the addition of DDAC disinfectant resulted in a reduction of more than 4 log_10_ CFU/carrier for all bacteria and textiles. The results showed that *L. monocytogenes* was the most susceptible to disinfection with DDAC, followed by *S. enterica* Typhimurium, while *B. cereus* was the least susceptible. A similar trend was observed for the textiles, with the highest reduction achieved on polyester microfibre, followed by cotton and cotton/linen (Fig. 2e). Washing with commercial detergent at 40 °C using antibacterial-finished kitchen towels resulted in values below the detection limit for *S. enterica* Typhimurium and *L. monocytogenes* on all three samples (the minimum detection limit is 1 CFU/mL); however, the required efficiency was not achieved for *B. cereus* (Fig. 2f). During the experiment we also monitored the pH and found a slight increase, for example 7.9 before washing, 8.1 after washing with detergent at 40 °C, and 8.2 after washing with detergent at 60 °C. 

**Fig. 3:**
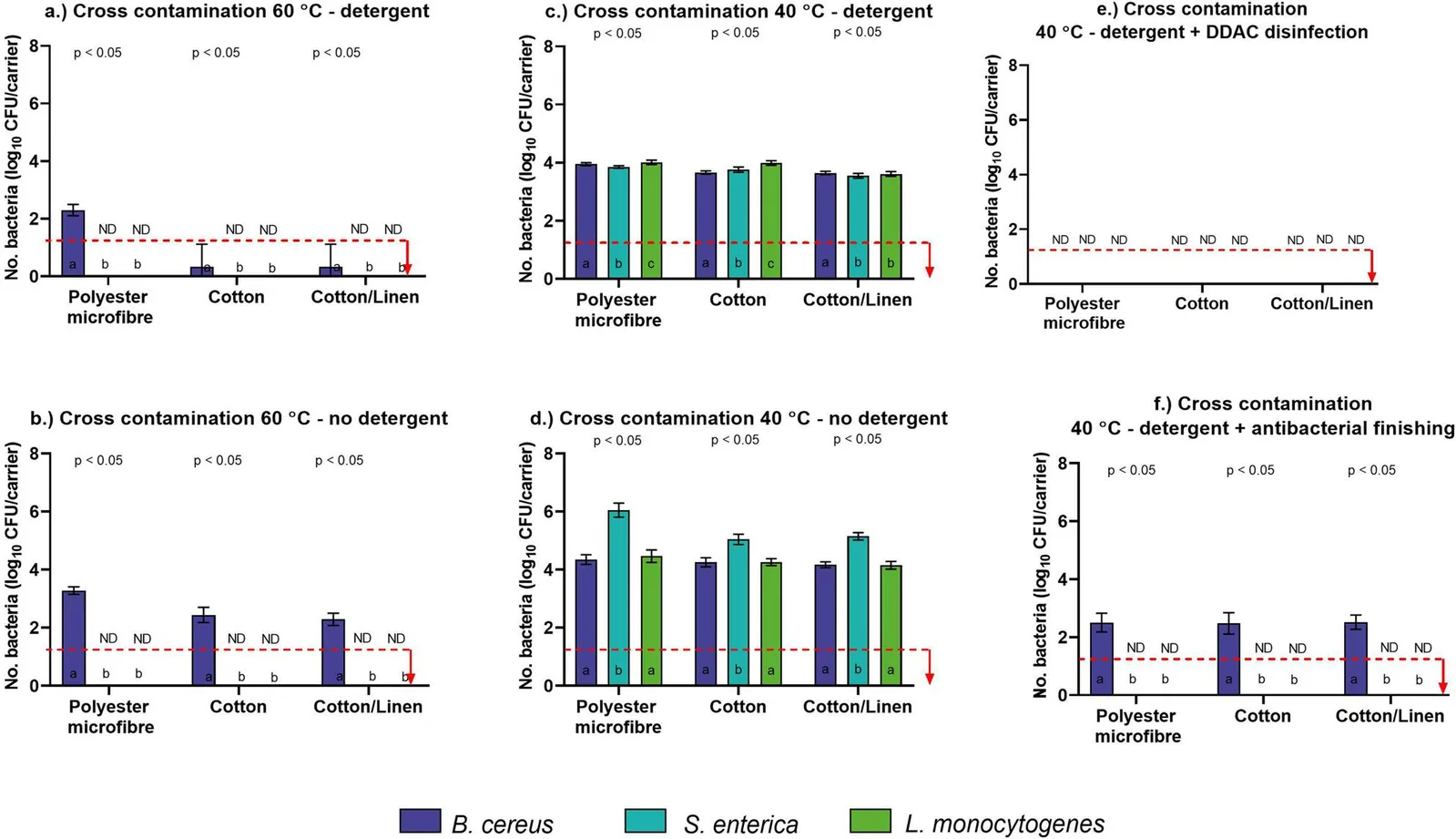
Cross-contamination of* B. cereus, S. enterica* Typhimurium, and* L. monocytogenes* (log10 CFU/carrier) on polyester microfibre, cotton, and cotton/linen after (**a**) washing with detergent at 60 °C, **b** washing without detergent at 60 °C, **c** washing with detergent at 40 °C, **d** washing without detergent at 40 °C, **e** washing at 40 °C with detergent and DDAC disinfectant, and **f** washing antibacterial-finished samples with detergent at 40 °C. ANOVA and Duncan's post hoc test. Different letters above the columns (**a**–**c**) indicate significant differences in Duncan's test (*p* < 0.05). ND – not detected. The red dashed line represents the ISO 17658 standard threshold for cross-contamination (≤1.5 log10 CFU/carrier)

However, washing can remove cells from textiles into the wash water, where they can contaminate other textiles or washing and drying appliances. Therefore, cross-contamination is an important factor when assessing laundry hygiene. The findings demonstrate low cross-contamination during washing with commercial detergent at 60 °C. Although we found cross-contamination below the detection limit for *S. enterica* Typhimurium and *L. monocytogenes* on all tested textiles, low cross-contamination was observed for *B. cereus* on cotton and cotton/linen. Notably, the highest cross-contamination (> 1.5 log_10_ CFU/carrier) was found for *B. cereus* on polyester microfibre (Fig. 3a). Similar results were seen for washing at 60 °C without detergent, with a higher cross-contamination rate for *B. cereus* on all textiles (Fig. 3b). Washing with commercial detergent at 40 °C resulted in high cross-contamination (> 3.7 log_10_ CFU/carrier) for all tested bacteria and textiles. A closer analysis shows similar bacterial loads for all strains, with the lowest cross-contamination observed for cotton/linen, followed by cotton and polyester microfibre, respectively (Fig. 3c). This effect of cross-contamination is even more pronounced when washing at 40 °C without detergent (Fig. 3d).

Furthermore, adding DDAC disinfectant to the washing process reduced bacterial counts of all tested strains on all textiles to below the detection limit, and cross-contamination was also below the detection limit (Fig. 3e). Washing antibacterial-finished samples with commercial detergent at 40 °C produced results similar to washing at 60 °C. Counts of *S. enterica* Typhimurium and *L. monocytogenes* were below the detection limit on polyester microfibre, cotton, and cotton/linen, while *B. cereus* cross-contamination remained more persistent (> 1.5 log_10_ CFU/carrier) (Fig. 3f). 

**Fig. 4:**
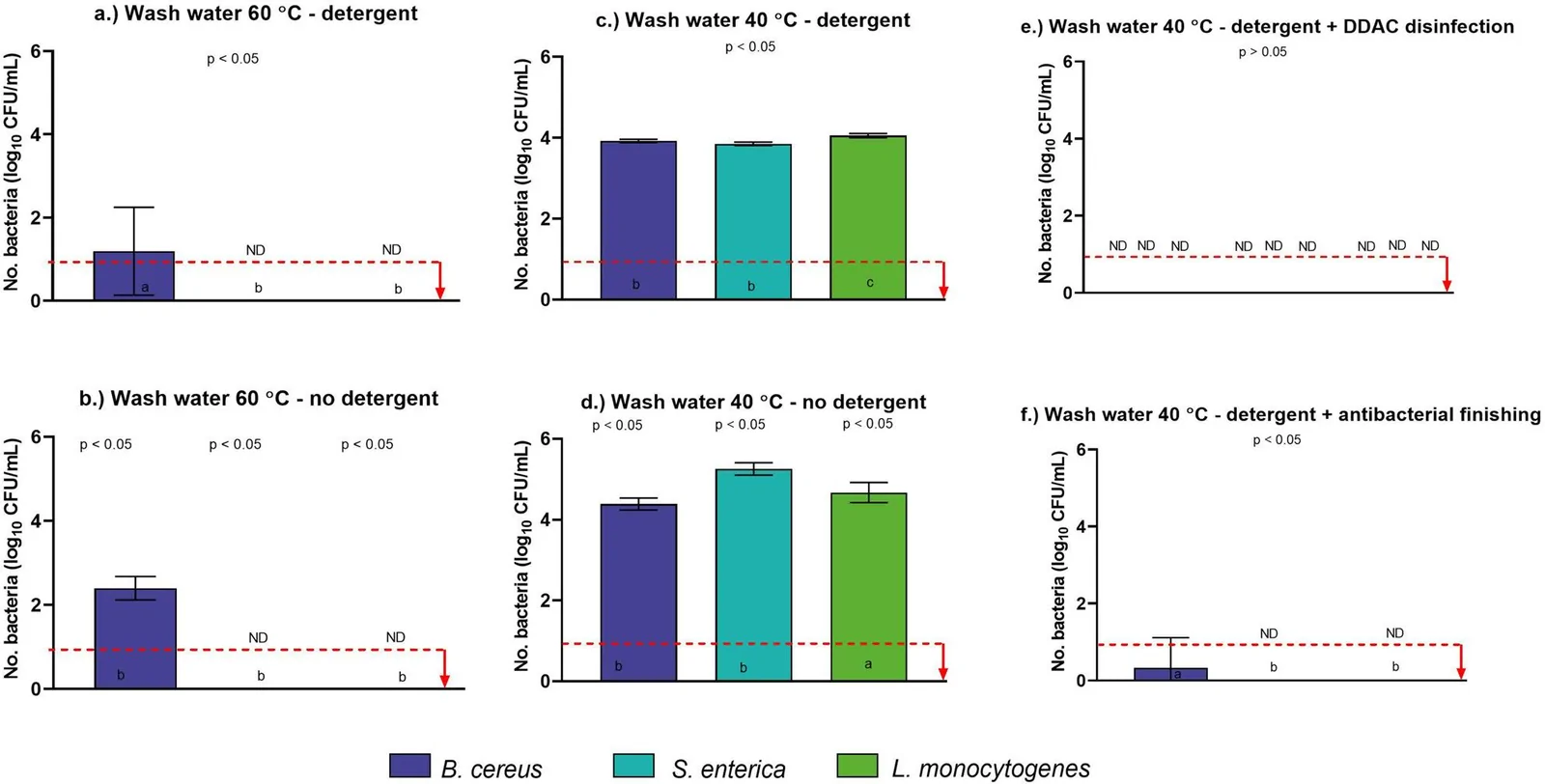
Wash water of *B. cereus*, *S. enterica *Typhimurium, and *L. monocytogenes* (log10 CFU mL− 1) on polyester microfibre, cotton, and cotton/linen after (**a**) washing with detergent at 60 °C, **b** washing without detergent at 60 °C, **c** washing with detergent at 40 °C, **d** washing without detergent at 40 °C, **e** washing at 40 °C with detergent and DDAC disinfectant, and **f** washing antibacterial-finished samples with detergent at 40 °C. ANOVA and Duncan’s post hoc test. Different letters above the columns (**a**–**c**) indicate significant differences in Duncan’s test (*p *< 0.05). ND – not detected. The red dashed line represents the ISO 17658 standard threshold for wash water (≤ 1.1 log10 CFU/carrier)

Subsequently, we analysed the bacterial counts in the wash water at the end of the washing cycle. The results revealed a similar pattern to that observed for cross-contamination. Washing at 60 °C with commercial detergent reduced *S. enterica* Typhimurium and *L. monocytogenes* in the wash water to below the detection limit, while *B. cereus* survived in the wash water when washing polyester microfibre (1.1 log_10_ CFU/mL), cotton (0.5 log_10_ CFU/mL), and cotton/linen (1.1 log_10_ CFU/mL) (Fig. 4a). A similar trend was observed for washing without detergent at 60 °C, although higher colony counts were detected for *B. cereus* (Fig. 4b).

Washing with commercial detergent alone did not eliminate bacteria in the wash water. Analysis of the data shows almost 4 log_10_ CFU/mL in the wash water for all tested bacteria when washing polyester microfibre, cotton, or cotton/linen, with the highest load for *L. monocytogenes* (Fig. 4c). In the experiment involving washing at 40 °C without detergent, even higher bacterial counts were detected (> 4 log_10_ CFU/mL), indicating a combined effect of temperature and detergent on bacteria (Fig. 4d). Adding DDAC disinfectant to the wash cycle with commercial detergent at 40 °C reduced *S. enterica* Typhimurium, *L. monocytogenes*, and *B. cereus* to below the detection limit on all tested textiles (Fig. 4e). The application of antibacterial finish to the kitchen towels studied produced similar results, except for *B. cereus*, for which a low bacterial count (0.5 log_10_ CFU/mL) was found in the wash water when washing polyester microfibre (Fig. 4f). 

## Discussion

Consumers’ risky habits in domestic kitchens can lead to foodborne outbreaks, as kitchen textiles come into direct contact with food. A study analysed the contamination of kitchen towels and found coliform bacteria, including Salmonella spp., on most textile samples [23]. Therefore, our study focused on analysing washing scenarios to mitigate the risk of domestic kitchen contamination with foodborne bacteria. More specifically, the study assessed the hygienic effectiveness of lowering domestic wash-water temperatures in line with trends towards environmental sustainability and highlighted the need to consider the required hygiene for each individual textile product. We found that washing at 60 °C with detergent was sufficient to eliminate all bacterial strains used, while the same washing conditions at 40 °C with detergent did not meet hygiene requirements. This concerning finding is due to the fact that the threshold temperature for the inactivation of non-sporulating bacteria was approximately 50 °C or higher [24–27]. Furthermore, high temperatures influenced the structural and physiological properties of the bacteria, specifically their membranes, RNA, DNA, ribosomes, proteins, and enzymes [25, 28]. Not only is the higher temperature itself significant, but its influence on the performance of the detergent is also considerable. The composition of the detergent used is specifically designed to work better in hot water, as the solubility of the ionic surfactant increases with temperature at a given pressure [29]. Our study demonstrated a much lower reduction in bacterial contamination when the textile was washed at 60 °C–40 °C without detergent, indicating a combined effect of temperature and detergent.

Among the bacterial strains tested, *B. cereus* showed the lowest reduction after washing at 60 °C, which can be attributed to the fact that bacteria of the genus *Bacillus* are difficult to remove regardless of the surface type [30]. Moreover, *B. cereus* can survive harsh environmental conditions in the form of spores, making the strains resistant to various disinfectants and heat [31, 32]. In addition, our study shows that washing at a lower temperature (40 °C) with a commercial detergent was not sufficient to provide adequate hygiene, regardless of the bacterial strain or textile material used. The highest reduction was, however, found on the polyester microfibre, which also has the most closed and dense structure, with fine filaments closely packed into three-dimensional surface elements. This accords with the finding that bacterial cells tend to adhere more to porous surfaces than to their denser counterparts [33]. This textile sample also has the highest surface roughness. Although rougher surfaces have been found to favour bacterial adhesion by providing more available areas for attachment [34], in our case the synergistic effect of surface roughness and hydrophobicity was evident, as the polyester microfibre has the lowest moisture content among the samples analysed. Cellulose fibres, including cotton and linen, are known for their hydrophilic properties, porous structure, and ability to bind water [35].

The combination of nanoscale roughness and hydrophobic surface chemistry hinders bacterial adhesion by reducing the effective contact area and creating energetically unstable adhesion sites [36]. The lowest Sku value of the polyester microfibre kitchen towel also indicates flat, plateau-like features, showing that the topography is less pronounced, with fewer sharp peaks or deep valleys, and therefore fewer depressions where bacterial cells can be protected from external forces [37–39]. Rating based on absorbency, and thus utility value, clearly places natural kitchen towels in first place; however, the hygiene of such towels is questionable. The ability to retain moisture is one of the characteristics that makes textile materials more prone to bacterial growth, creating an ideal environment for microbes by providing oxygen, water, and warmth [40]. This characteristic is partly due to the chemical composition of the fibres [41], which in this study was analysed using FTIR. The spectrum of the polyester microfibre kitchen towel clearly shows a substantially lower peak at 3330 cm⁻¹ than the other two textile materials, corresponding to the O–H band, a functional group that determines a material’s tendency to interact with water molecules. In contrast with synthetic fibre, both cotton and cotton/linen have an abundance of free hydroxyl groups, which explains their higher hydrophilicity and the favourable conditions for the growth and reproduction of *B. cereus*, *S. enterica* Typhimurium, and *L. monocytogenes*.

When washing temperatures were reduced to 40 °C, it was clearly observed that the addition of DDAC disinfectant caused a reduction, regardless of the bacterial strain or textile material used. The primary disinfecting compound in the DDAC disinfectant is didecyldimethylammonium chloride, while three other compounds – methylisothiazolinone, phenoxyethanol, and 2-bromo-2-nitropropane-1,3-diol – act as bacterial preservatives. DDAC is commonly found in healthcare product formulations and household cleaners, as well as in disinfectants for surfaces and equipment used in food and feed areas [42–44]. Its antimicrobial action, exhibiting both bacteriostatic and bactericidal activity on bacterial membranes, depends on its concentration and the growth phase of the population [45, 46]. DDAC was most effective against Gram-positive *L. monocytogenes*, followed by Gram-negative *S. enterica* Typhimurium, and finally Gram-positive *B. cereus*. Other studies also show high antibacterial efficiency of DDAC disinfectant against *S. enterica* Typhimurium [47] and *B. cereus* [48], supporting the high antimicrobial efficacy observed in the present study.

Following the reduction of bacterial strains on the three textiles, the trend for washing at 40 °C differed slightly from that at 60 °C; the greatest reduction was observed on polyester microfibre, followed by cotton and then cotton/linen. These findings are supported by our results on moisture content, with the lowest moisture content on microfibre, followed by cotton and cotton/linen, respectively. Our study also demonstrated that a lower washing temperature reduced the activation of surfactant substances in the detergent used, and thus the inherent hydrophobic properties became more prominent. Finally, the active compound in the finishing agent used, benzalkonium chloride, is known to inhibit biofilm formation [49], as its action involves perturbing and disrupting the cell membrane [50]. However, while it was intended to reduce *S. enterica* Typhimurium and *L. monocytogenes*, it was again less effective against *B. cereus*. In line with our findings, one study found that benzalkonium chloride disinfection is less efficient in controlling *B. cereus* in aquatic product processing [51]. Furthermore, it is important to consider that the observed decline in bacterial counts might not solely reflect cell death. The strains may have entered alternative physiological states, such as the viable but non-culturable (VBNC) state or stress-induced dormancy. In these states, bacteria remain metabolically active but fail to form colonies on standard growth media, which can lead to an underestimation of the actual bacterial population when relying exclusively on plate-culturing techniques [52].

Bacterial presence on kitchen towels is critical not only as a direct source of infection, but also because they can act as microbial reservoirs and serve as sources of further cross-contamination. For example, one study found that bacteria at room temperature survived for up to 206 days on polyester, and up to 90 days on cotton and mixed fibres [53]. We demonstrated that after washing at 60 °C with commercial detergent, low cross-contamination of *B. cereus* was found on natural textile materials (specifically cotton and cotton/linen), while the highest cross-contamination was observed on polyester microfibre. In another study, the transfer efficiency of *Escherichia coli*, *Staphylococcus aureus*, and *Bacillus thuringiensis* was greater on non-porous surfaces than on porous surfaces [54]. This is consistent with our results, as the polyester kitchen towel also exhibited a more closed and dense structure with lower surface openness, although we tested cross-contamination in the washing bath. Another textile characteristic that influences bacterial transfer is surface roughness. One study reported that the roughness of polypropylene fabric discouraged the transfer of bacterial cells, while the highest cell transfer was observed for viscose and polyester fabrics, which had the least surface roughness [55]. This contradicts our findings, as cotton and cotton/linen exhibit lower surface roughness than the polyester microfibre kitchen towel. However, it is important to note that in our study, measurements of surface roughness took into account a broader area of the textile sample, thus providing information on surface roughness at the yarn or weave level. Furthermore, the SEM images from our study revealed that the smoothest fibre surface belonged to the synthetic kitchen towel, while the cotton and cotton/linen fibres, due to their natural origin, showed a more textured and uneven surface.

Moreover, the study correlated the effect of surface roughness and bacterial transfer with moisture regain, which is highest for viscose, followed by polyester, and finally polypropylene [56]. Other studies have also found that increasing moisture results in higher cell transfer proportions, regardless of whether the material is textile or another type [57–60]. In another study, it was suggested that the thickness of the textile samples may be the reason for bacterial regrowth [12]. This is also stated in our article, supporting the view that polyester microfibre (with the greatest measured thickness) may represent a potentially critical surface for bacterial strains. Because bacteria are transferred from one surface to another, it is also recommended that the use of 100% polyester in healthcare settings be minimised [61]. During washing, both inoculated and non-inoculated textile samples (i.e. donor and recipient fabrics) are wet, which, according to research by Varshney et al., is one of the main reasons for the highest transfer of bacterial cells compared with other conditions in which donor and recipient fabrics are tested [55].

The synergistic effect of the hydrophobicity of both the textile material and the bacterial strain is also noteworthy, as polyester microfibre and *B. cereus* were both found to be hydrophobic. In our other study, the hydrophobic nature of both polyester and methicillin-resistant *Staphylococcus aureus* (MRSA) probably contributes to their high affinity for bacterial cross-contamination [62]. Bacterial cross-contamination was significantly greater and more apparent when washing at a lower temperature (40 °C) with detergent, possibly due to reduced activation of the active compound. Cross-contamination was even more pronounced when no detergent was used in the experiment, indicating that chemicals in detergent play an important role in reducing the transfer of bacteria from wash water to textiles. It should also be noted that standard household detergents are designed primarily to remove dirt and stains, not to disinfect [63]. Comparing the effectiveness of detergent combined with DDAC disinfectant with that of detergent combined with antibacterial finishing showed lower bacterial transmission in the former. DDAC disinfectant prevents the transfer of bacterial cells from donor to recipient fabrics by deactivating the bacterial cells in the wash water, whereas antibacterial finishing acts more locally on the fibre surface, in direct contact with the cells. The combination of detergent and DDAC disinfectant was found to be the most effective overall against bacterial cross-contamination. For example, a study reported that at low washing temperatures (*E. coli*,* S. aureus*,* P. aeruginosa*), hygiene can be achieved only if an antimicrobial agent is added to the washing process [64].

The results of the bacterial counts in the wash water showed a pattern similar to that observed for cross-contamination. These findings indicate a correlation: the higher the number of bacterial cells in the wash water, the greater the likelihood of cross-contamination of non-inoculated samples, which poses a high risk of food poisoning. Strains present in the wash water are not only a risk for cross-contamination of specific textile materials, but can also form biofilms inside washing machines, thereby mediating contamination of subsequent laundry [15, 65]. From an environmental perspective, there is a tendency to reuse wash water to reduce water consumption, as in European countries approximately 60 L of freshwater is used per washing cycle [66]. This increasing interest in water reuse also highlights the need for environmentally friendly disinfectants that effectively inactivate microorganisms while minimizing ecological impact. Although DDAC-based disinfectants can substantially improve hygiene during low-temperature washing, the environmental profile of disinfectants should be carefully considered. Hydrogen peroxide-based disinfectants represent one promising alternative due to their decomposition into water and oxygen, leaving no persistent residues [67]. However, the development of stable and sufficiently effective formulations, particularly at low hydrogen peroxide concentrations, remains a technological challenge because of hydrogen peroxide instability and the need to maintain antimicrobial efficacy during storage and use [67]. Failure to deactivate bacteria in wash water can result in infection and the transfer of resistance genes to the environment. According to the results, DDAC disinfectant alternatives provide antibacterial finishing; however, their effectiveness is more limited and depends on the type of microorganism and their specific application for achieving textile hygiene, while the wash water after such washing remains full of bacterial cells and is therefore less suitable for reuse. Overall, the results highlight the need for a thorough understanding of the interacting factors involved in the washing process, indicating that eliminating or reducing one significant factor requires a strong substitute to achieve adequate hygiene without negative environmental impact.

## Conclusions

Low-temperature household laundering of kitchen towels contaminated with bacteria such as *B. cereus*, *S. enterica* Typhimurium, and *L. monocytogenes* poses a significant risk of foodborne illness outbreaks. This study shows that the hygienic safety of kitchen towels depends not only on washing conditions but also on the inherent resilience of the microorganisms and the characteristics of the textile materials. We found that typical kitchen towels made of cotton, cotton/linen, or polyester differ significantly in their surface properties, despite serving the same purpose. Cotton and cotton/linen have similar structures, whereas polyester exhibits a more complex topography. A similar pattern was observed for surface openness: polyester has a closed, dense structure compared with cotton, which is more open and air-permeable. The same trend is seen in their ability to bind water: cotton/linen has the highest moisture content, followed by cotton, while polyester microfibre absorbs the least water.

All this indicates that the surface characteristics of kitchen towels govern bacterial adhesion and the effectiveness of washing. Overall, the findings underline that relying on detergent alone, especially at lower temperatures, offers insufficient protection against microbial survival and cross-contamination. In particular, the persistence of *B. cereus* across several laundering conditions highlights the challenge posed by spore-forming bacteria and their ability to withstand both thermal and chemical stress. From a hygiene perspective, washing temperature and detergent have a combined effect on bacterial reduction, cross-contamination and wash water contamination, as the addition of detergent reduced bacterial counts for all tested bacterial strains and textiles. The results collectively indicate that effective hygiene can be achieved either through sufficiently high washing temperatures or by incorporating disinfectant agents into the laundering process or antimicrobial functionalised textile products. Special care should be taken when using disinfectant agents in laundry to ensure they have only a minimal impact on the environment while still achieving an adequate reduction in micro-organisms. Disinfectants such as DDAC substantially enhance the safety margin during low-temperature washing, demonstrating their value in compensating for energy-saving wash cycles. The advantage of adding disinfectant to the washing cycle is that it reduces the risk of cross-contamination of textiles and contamination of wash water, and consequently of washing and drying appliances, whereas antibacterial finishing acts more locally on the fabric surface.

Antibacterial finishes show promise as an additional strategy, although their efficacy remains organism-dependent and may require further refinement for reliable control of resistant species. Textile composition also plays a significant role in determining hygienic outcomes, indicating that material selection should be considered in applications where repeated contamination is likely. Synthetic microfibre, cotton, and cotton/linen each interact differently with bacteria and cleaning agents, affecting both microbial reduction and cross-contamination potential. Taken together, these insights highlight the need for tailored laundering strategies that account for temperature, chemistry, and material design to reliably prevent microbial transmission in kitchen environments. The study emphasises that low-temperature washing alone cannot guarantee hygiene and that integrating environmentally friendly disinfection or advanced textile technologies is essential to mitigate the public health risks associated with contaminated kitchen towels.

## Data Availability

The data supporting the findings of this study, including the final visual representations, are provided in the manuscript and the Supplementary Material. The raw data are available from the corresponding author upon reasonable request.

## Abbreviations

B. cereus: Bacillus cereus
S. enterica Typhimurium: Salmonella enterica serovar Typhimurium
L. monocytogenes: Listeria monocytogenes
DDAC: Didecyldimethylammonium chloride
TSA: Tryptic Soy Agar
SEM: Scanning electron microscopy
CLSM: Confocal laser scanning microscope
3D: Three-dimensional
FTIR: Fourier transform infrared spectroscopy
MATH: Microbial adhesion to hydrocarbons
PBS: Phosphate-buffered saline
BSA: Bovine serum albumin
SBL: Soil Ballast Load
ANOVA: One-way analysis of variance
MRSA: Methicillin-resistant Staphylococcus aureus
